# Prognostic value of tumor markers and ctDNA in patients with resectable gastric cancer receiving perioperative treatment: results from the CRITICS trial

**DOI:** 10.1007/s10120-021-01258-6

**Published:** 2021-10-29

**Authors:** Astrid E. Slagter, Marieke A. Vollebergh, Irene A. Caspers, Johanna W. van Sandick, Karolina Sikorska, Pehr Lind, Marianne Nordsmark, Hein Putter, Jeffrey P. B. M. Braak, Elma Meershoek-Klein Kranenbarg, Cornelis J. H. van de Velde, Edwin P. M. Jansen, Annemieke Cats, Hanneke W. M. van Laarhoven, Nicole C. T. van Grieken, Marcel Verheij

**Affiliations:** 1 Department of Radiation Oncology, Netherlands Cancer Institute, Amsterdam, The Netherlands; 2 Department of Gastrointestinal Oncology, Netherlands Cancer Institute, Amsterdam, The Netherlands; 3 Department of Surgery, Netherlands Cancer Institute, Amsterdam, The Netherlands; 4 Department of Biometrics, Netherlands Cancer Institute, Amsterdam, The Netherlands; 5 Department of Oncology, Stockholm Söder Hospital, Stockholm, Sweden; 6 Karolinska Institutet, Stockholm, Sweden; 7 Department of Medical Oncology, Aarhus University, Aarhus, Denmark; 8 Department of Biometrics, Leiden University Medical Center, Leiden, The Netherlands; 9 Department of Surgery, Leiden University Medical Center, Leiden, The Netherlands; 10 Department of Medical Oncology, Amsterdam University Medical Centers, University of Amsterdam, Amsterdam, The Netherlands; 11 Department of Pathology, Amsterdam University Medical Centers, Cancer Center Amsterdam, Amsterdam, The Netherlands; 12 Department of Radiation Oncology, Radboud University Medical Center, Geert Grooteplein 32, 6500 HB Nijmegen, The Netherlands

**Keywords:** Tumor markers, CEA, CA 19-9, ctDNA, Resectable gastric cancer

## Abstract

**Aim:**

To evaluate the prognostic value of tumor markers in a European cohort of patients with resectable gastric cancer.

**Methods:**

We performed a post hoc analysis of the CRITICS trial, in which 788 patients received perioperative therapy. Association between survival and pretreatment CEA, CA 19-9, alkaline phosphatase, neutrophils, hemoglobin and lactate dehydrogenase were explored in uni- and multivariable Cox regression analyses. Likelihoods to receive potentially curative surgery were investigated for patients without elevated tumor markers versus one of the tumor markers elevated versus both tumor markers elevated. The association between tumor markers and the presence of circulating tumor DNA (ctDNA) was explored in 50 patients with available ctDNA data.

**Results:**

In multivariable analysis, in which we corrected for allocated treatment and other baseline characteristics, elevated pretreatment CEA (HR 1.43; 95% CI 1.11–1.85, *p* < 0.001) and CA 19-9 (HR 1.79; 95% CI 1.42–2.25, *p* < 0.001) were associated with worse OS. Likelihoods to receive potentially curative surgery were 86%, 77% and 60% for patients without elevated tumor marker versus either elevated CEA or CA 19-9 versus both elevated, respectively (*p* < 0.001). Although both preoperative presence of ctDNA and tumor markers were prognostic for survival, no association was found between these two parameters.

**Conclusion:**

CEA and CA 19-9 were independent prognostic factors for survival in a large cohort of European patients with resectable gastric cancer. No relationship was found between tumor markers and ctDNA. These factors could potentially guide treatment choices and should be included in future trials to determine their definitive position.

**Trial registration:**

ClinicalTrial.gov identifier: NCT00407186. EudraCT number: 2006-00413032.

**Supplementary Information:**

The online version contains supplementary material available at 10.1007/s10120-021-01258-6.

## Introduction

In the western world, gastric cancer is usually diagnosed at an advanced stage [1]. In Europe, the standard treatment for patients with locoregional gastric cancer consists of resection with D2 lymph node dissection combined with perioperative chemotherapy [2]. Other evidence-based treatment options include pre- or postoperative chemoradiotherapy or postoperative chemotherapy [2–6]. Currently, there are no diagnostic tests to guide treatment choice for above-mentioned different treatment modalities.

Both carcinoembryonic antigen (CEA) and carbohydrate antigen 19-9 (CA 19-9) are well-known tumor markers and have been evaluated for their prognostic value in gastric cancer in large meta-analyses [7, 8]. Elevated CEA or CA 19-9 levels have been associated with worse outcome in terms of overall survival (OS) and recurrence. Although these large meta-analyses provide important information, most studies on tumor markers have been carried out in Asia, and/or included patients with metastatic gastric cancer. Results in Asian gastric cancer patients cannot be translated directly to European gastric cancer patients since there are well-known clinicopathological differences [9–12]. The prognostic value of preoperative CEA and CA 19-9 levels in gastric cancer patients in European countries is still largely unknown.

Other blood-derived laboratory parameters which have been studied as prognostic markers in patients with gastric cancer include neutrophil counts, hemoglobin, alkaline phosphatase and lactate dehydrogenase. High neutrophil counts [13] and low hemoglobin levels [14], high alkaline phosphatase [15] and high serum lactate hydrogenase [16–18] were found to be associated with a poorer OS. However, most of these studies included small numbers of patients and/or included patients with metastatic gastric cancer.

In addition to the classical serological tumor markers, circulating tumor DNA (ctDNA) has recently emerged as a potential prognostic marker in various tumor types including patients with gastric cancer. The presence of ctDNA after preoperative treatment and postoperatively has been shown to be associated with recurrence and worse overall survival among 50 patients who participated in the CRITICS trial [19]. The presence of pretreatment ctDNA alone was not associated with survival in a subset of 50 patients. However, ctDNA ‘response’ (i.e., ctDNA detectable before start of treatment but undetectable preoperatively) was associated with complete or near complete histological response.

Ideally, combining various prognostic markers at crucial pretreatment time-points would assist clinical decision making and guide ongoing therapies. The aim of this study was to determine the prognostic value of hemoglobin, neutrophils, alkaline phosphatase, lactate dehydrogenase, CEA and CA 19-9 levels before start of preoperative chemotherapy in a European patient population with gastric cancer treated with curative intent. For this purpose, we studied patients who were included in the phase III CRITICS trial [20].

## Methods

### Patient selection

**Fig. 1:**
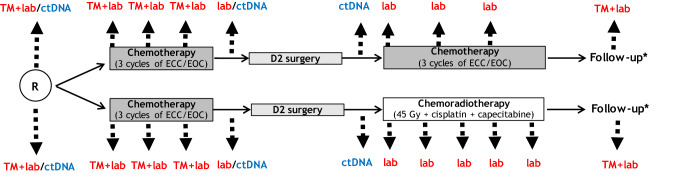
Overview of CRITICS study design including time-points of blood samples. *ctDNA* circulating tumor DNA, *ECC* epirubicin/cisplatin/capecitabine, *EOC* epirubicin/oxaliplatin/capecitabine, *lab* laboratory parameters (neutrophil count, hemoglobin, alkaline phosphatase and lactate dehydrogenase), *TM* tumor markers (CEA and CA 19-9). *Every month until 3 months after surgery, every 3 months until 1 year after surgery, 6 months until 5 years after surgery

The CRITICS trial was a phase III open label trial in which 788 patients with stage IB-IVA (non-metastatic) (AJCC 6th edition) adenocarcinoma of the stomach or gastro-esophageal junction (GEJ) were included. Patients were randomized before start of preoperative therapy to pre- plus postoperative chemotherapy or preoperative chemotherapy plus postoperative chemoradiotherapy [21]. All patients were intended to receive resection plus D2 lymph node dissection. The following baseline characteristics were available: age, gender, WHO performance status, Lauren classification, tumor localization, body-mass index (BMI) and allocated treatment. Results of blood-derived laboratory parameters were recorded before any treatment, before every chemotherapy course, postoperatively and during follow-up) (Fig. 1). Blood samples for ctDNA determination were taken prior to start of preoperative chemotherapy, after three cycles of preoperative chemotherapy and postoperatively in a subset of patients (*n* = 50).

### Tumor markers and other blood-derived laboratory parameters

All tests were performed in certified laboratories according to local standards. The following cutoff values were used as upper limit of normal (ULN): CEA ≤ 6 µg/L, CA 19-9 ≤ 37 kU/L, alkaline phosphatase ≤ 115 U/L, neutrophils > 7.5 10^9^/L, hemoglobin > 10 mmol/L for females or > 11 mmol/L for males and LDH ≤ 248 U/L. CRITICS inclusion criteria included: alkaline phosphatase ≤ 3 times ULN, neutrophils ≥ 1.5 10^9^/L and hemoglobin ≥ 5 mmol/L. For the other factors, no in-/exclusion criteria were applicable. Methods and results of ctDNA analyses were published previously [19].

### Statistical analysis

All data were analyzed using IBM SPSS statistics version 25. Continuous variables were presented as medians plus interquartile ranges (IQR) and categorical variables were presented as frequencies plus percentages. Differences between groups were tested using Chi-square test, Fisher’s exact test or Mann–Whitney *U* test as appropriate. Survival curves were produced using the Kaplan–Meier method and compared using the log-rank test. Hazard ratios were generated using Cox proportional hazards regression. The level for significance was set at *p* < 0.05. Event-free survival was defined as time from randomization until event or last follow-up. An event was defined as local/regional or distant progression or recurrence or death from any cause. Overall survival was defined as time from randomization until death or last follow-up. Data-lock was June 2021.

Baseline characteristics were displayed for CEA within and higher than ULN and for CA 19-9 within and higher than ULN. Likelihoods for receiving surgery with curative intent (R0 or R1 resection) were compared for patients who had both tumor markers (CEA and CA 19-9) within ULN versus one of the tumor markers elevated, versus both tumor markers elevated. We evaluated whether elevated levels of tumor markers (both pretreatment as prior to cycle 3 of preoperative chemotherapy) were associated with OS and EFS. Univariable Cox regression analysis was performed to evaluate the relationship between laboratory parameters and survival, other baseline characteristics were also included. Variables with a *p* value of < 0.1 and allocated treatment were explored in multivariable analysis. Binominal values were tested in univariable analysis rather than continuous variables, because this is more convenient in clinical practice. For factors significant in multivariable analysis, linear relationships were tested using Cox regression analysis. In addition, we evaluated relationship between difference of tumor markers during therapy and survival. The difference between pretreatment and cycle 3 was evaluated categorically: (1) both pretreatment and at cycle 3 levels ≤ ULN, (2) pretreatment ≤ ULN increased to > ULN at cycle 3, (3) pretreatment > ULN decreased to ≤ ULN at cycle 3, or (4) both pretreatment and at cycle 3 levels > ULN. Lastly, we evaluated the association between ctDNA itself and in combination with tumor markers.

## Results

Pretreatment blood-derived laboratory parameters were available in the majority of the 788 patients, including CEA for 725 patients (92%) and CA 19-9 for 699 patients (89%).

### Baseline characteristics

Baseline characteristics were compared for patients who had pretreatment CEA and CA 19-9 levels within and higher than ULN (Table 1). For patients who had a pretreatment CEA > ULN, median CEA level was 15.2 (IQR 8.85–43.05) µg/L; for patients who had a pretreatment CA 19-9 > ULN, median CA 19-9 level was 133.5 (IQR 61.25–418.5) kU/L.

**Table 1:** Baseline characteristics

Variable	CEA ≤ ULN *N* = 604	CEA > ULN *N* = 121	*p* value	CA 19.9 ≤ ULN *N* = 531	CA 19.9 > ULN *N* = 168	*p* value
CEA/CA 19-9 level in µg/L/kU/L Median (range)	CEA level 2 (0–1533)			CA 19-9 level 11 (0–15,429)		
Age in years			0.987			0.182
< 60	243 (40%)	48 (40%)		222 (42%)	61 (36%)	
60–69	230 (38%)	46 (38%)		201 (38%)	62 (37%)	
≥ 70	131 (22%)	27 (22%)		108 (20%)	45 (27%)	
Sex			0.001			
Male	391 (65%)	97 (80%)		356 (67%)	117 (70%)	
Female	213 (35%)	24 (20%)		175 (33%)	51 (30%)	0.530
WHO PS			0.004			0.004
Missing	33	8		25	15	
0	420 (74%)	68 (60%)		375 (74%)	95 (62%)	
1	151 (26%)	45 (40%)		131 (25%)	58 (38%)	
Lauren classification (biopsy)			0.009			0.048
Intestinal	177 (29%)	51 (42%)		164 (31%)	56 (33%)	
Diffuse	195 (32%)	23 (19%)		170 (32%)	38 (23%)	
Mixed	35 (6%)	7 (6%)		26 (5%)	15 (9%)	
Unknown	197 (33%)	40 (33%)		171 (32%)	59 (35%)	
Tumor localization			< 0.001			0.002
GEJ	95 (16%)	37 (31%)		84 (16%)	44 (26%)	
Proximal	119 (20%)	28 (23%)		104 (20%)	41 (24%)	
Middle	190 (32%)	23 (19%)		159 (30%)	43 (26%)	
Distal	200 (33%)	33 (27%)		184 (35%)	40 (24%)	
BMI			0.084			0.543
≥ 30	84 (14%)	14 (12%)		69 (13%)	23 (14%)	
25–30	220 (36%)	39 (32%)		187 (35%)	63 (38%)	
18.5–25	289 (48%)	61 (50%)		264 (50%)	76 (45%)	
≤ 18.5	11 (2%)	7 (6%)		11 (2%)	6 (4%)	
BMI			0.028			0.985
Median (IQR)	25 (23–28)	25 (22–27)		25 (23–28)	25 (23–28)	
Allocated treatment			0.703			0.011
Postop CT	303 (50%)	63 (48%)		253 (48%)	99 (59%)	
Postop CRT	301 (50%)	58 (52%)		278 (52%)	69 (42%)	
CA 19-9 or CEA level*	Missing *n* = 28	Missing *n* = 6	< 0.001	Missing *n* = 7	Missing *n* = 2	< 0.002
≤ ULN	461 (80%)	63 (55%)		461 (88%)	114 (69%)	
> ULN	114 (20%)	52 (45%)		63 (12%)	52 (31%)	

*CA 19-9* carbohydrate antigen 19-9, *CEA* carcinoembryonic antigen, *postop* postoperative, *CRT* chemoradiotherapy, *CT* chemotherapy

^*^ULN for CEA was 6 µg/L, ULN for CA 19-9 was ≤ 37 kU/L

### Impact of pretreatment tumor markers on surgery

The likelihood of receiving surgery with curative intent was reduced for patients with elevated pretreatment tumor markers: 86% for patients who had both tumor markers within ULN versus 77% for patients who had either CEA or CA 19-9 elevated versus 60% in patients with both tumor markers elevated (*p* < 0.001). The proportions of patients who underwent palliative surgery were 9% for patients who had both tumor markers within ULN versus 15% for patients in whom either elevated CEA or CA 19-9 versus 31% for patients in whom both tumor markers were elevated prior to treatment.

### Prognostic value of blood-derived laboratory parameters

**Fig. 2:**
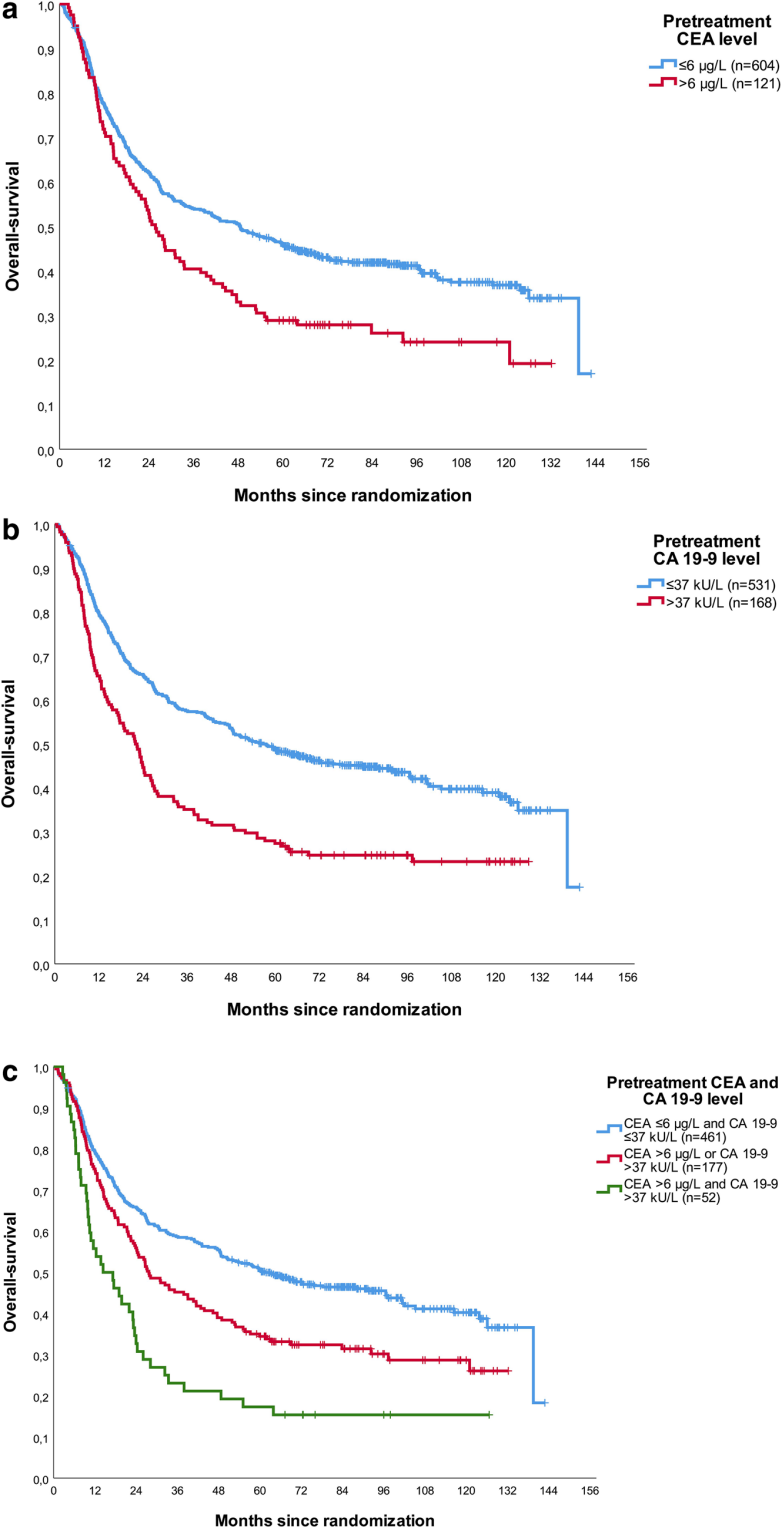
Overall survival curves for patients subdivided by pretreatment tumor markers. Figure 1a CEA (*p* = 0.002); Fig. 1b CA 19-9 (*p* < 0.001); Fig. 1c combination CEA and CA 19-9 (*p* < 0.001)

Median follow-up time for the entire cohort was 7.2 years. Figure 2 shows OS curves of patients stratified per pretreatment values of CEA, CA 19-9 and the combination. Elevated CEA (Fig. 2a) and CA 19-9 levels (Fig. 2b) were significantly associated with shorter OS. Five-year OS was 25% for patients with an elevated baseline CEA compared to 43% for patients with a baseline CEA within the ULN. For CA 19-9, 5-year survival was 25% for patients with an elevated level compared to 46% for patients with a CA 19-9 within the ULN. An even stronger relationship between tumor markers and OS was observed if both tumor markers were combined (Fig. 2c). Five-year OS was 11% versus 32% versus 48% for patients with both tumor markers elevated at baseline, either elevated CEA or CA 19-9 or both tumor markers within ULN, respectively. Similar distributions were observed for EFS (supplementary Fig. 1). Comparable survival curves were obtained of patients stratified by categories of tumor markers prior to cycle 3 of preoperative chemotherapy (supplementary Figs. 2 and 3).

Univariable analysis revealed elevated pretreatment CEA and CA 19-9 to be associated with OS and EFS (supplementary Tables 1 and 2). A regular Cox regression model was not appropriate to investigate if a linear relationship existed between the level of CEA/CA 19-9 and OS. To correct for extreme values of CEA and CA 19-9, log10 transformation of the data was performed. After transformation of the data, a linear relationship was observed between CEA and OS, with a HR of 1.25 (95% CI 1.06–1.47) (*p* = 0.010). Also for CA 19-9, a linear relationship was confirmed with a HR of 1.44 (95% CI 1.28–1.62) (*p* < 0.001).

In multivariable analysis, in which we corrected for allocated treatment and other baseline characteristics, both CEA and CA 19-9 revealed to be independent prognostic factors for OS (Table 2). A baseline CEA > ULN was associated with a worse OS with a HR of 1.43 (95% CI 1.11–1.85) (*p* = 0.007), and an elevated baseline CA 19-9 was associated with worse OS with a HR of 1.79 (95% CI 1.42–2.25) (*p* < 0.001). Multivariable analysis for EFS showed comparable results (supplementary Table 3).

**Table 2:** Multivariable analysis for prognostic factors on overall survival

Variable	*n* = 650	HR	95% CI	*p* value
Lauren classification				
Intestinal	200 (31%)	*		
Diffuse	194 (30%)	1.99	1.53–2.59	< 0.001
Mixed	37 (6%)	0.95	0.57–1.57	0.826
Unknown	219 (34%)	1.45	1.12–1.71	0.005
WHO PS				
0	466 (72%)	*		
1	184 (28%)	1.38	1.12–1.71	0.003
BMI				
≥ 30	87 (13%)	*		
25–30	235 (36%)	0.98	0.70–1.37	0.883
18.5–25	312 (48%)	1.29	0.93–1.77	0.124
≤ 18.5	16 (2%)	1.07	0.55–2.08	0.843
Allocated treatment				
Postop CT	395 (50%)	*		
Postop CRT	393 (50%)	1.06	0.87–1.30	0.545
CEA	Missing *n* = 138			
≤ 6 µg/L	543 (84%)	*		
> 6 µg/L	107 (16%)	1.43	1.11–1.85	0.007
CA 19-9	Missing *n* = 138			
≤ 37 kU/L	499 (77%)	*		
> 37 kU/L	151 (23%)	1.79	1.42–2.25	< 0.001

### Prognostic value of ‘serological response’

**Fig. 3:**
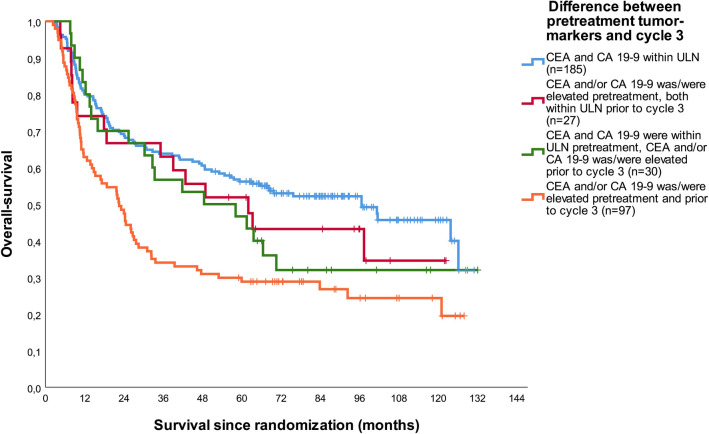
Overall survival curves for patients subdivided by change between pretreatment tumor markers and tumor markers prior to cycle 3 (*p* < 0.001)

For a total of 339 patients, tumor marker levels were available both pretreatment and prior to cycle 3 of preoperative chemotherapy. As shown in Fig. 3, patients who had both tumor markers within ULN at both time-points had the best OS. Patients who had elevated tumor marker(s) at the pretreatment time-point, which normalized to < ULN prior to cycle 3 had substantially worse survival with a HR of 1.27 (95% CI 0.75–2.16, *p* = 0.379). Patients who had normal tumor marker(s) pretreatment but elevated tumor marker(s) prior to cycle 3 of chemotherapy had comparable OS to the previous mentioned group with a HR of 1.39 (95% CI 0.85–2.25, *p* = 0.0.187). The group who had both elevated tumor marker(s) pretreatment and prior to the third cycle of chemotherapy had the worst OS with a HR of 2.02 (95% CI 1.48–2.75, *p* < 0.001). A similar pattern was observed using EFS as outcome (supplementary Fig. 4).

### Tumor markers and ctDNA

**Fig. 4:**
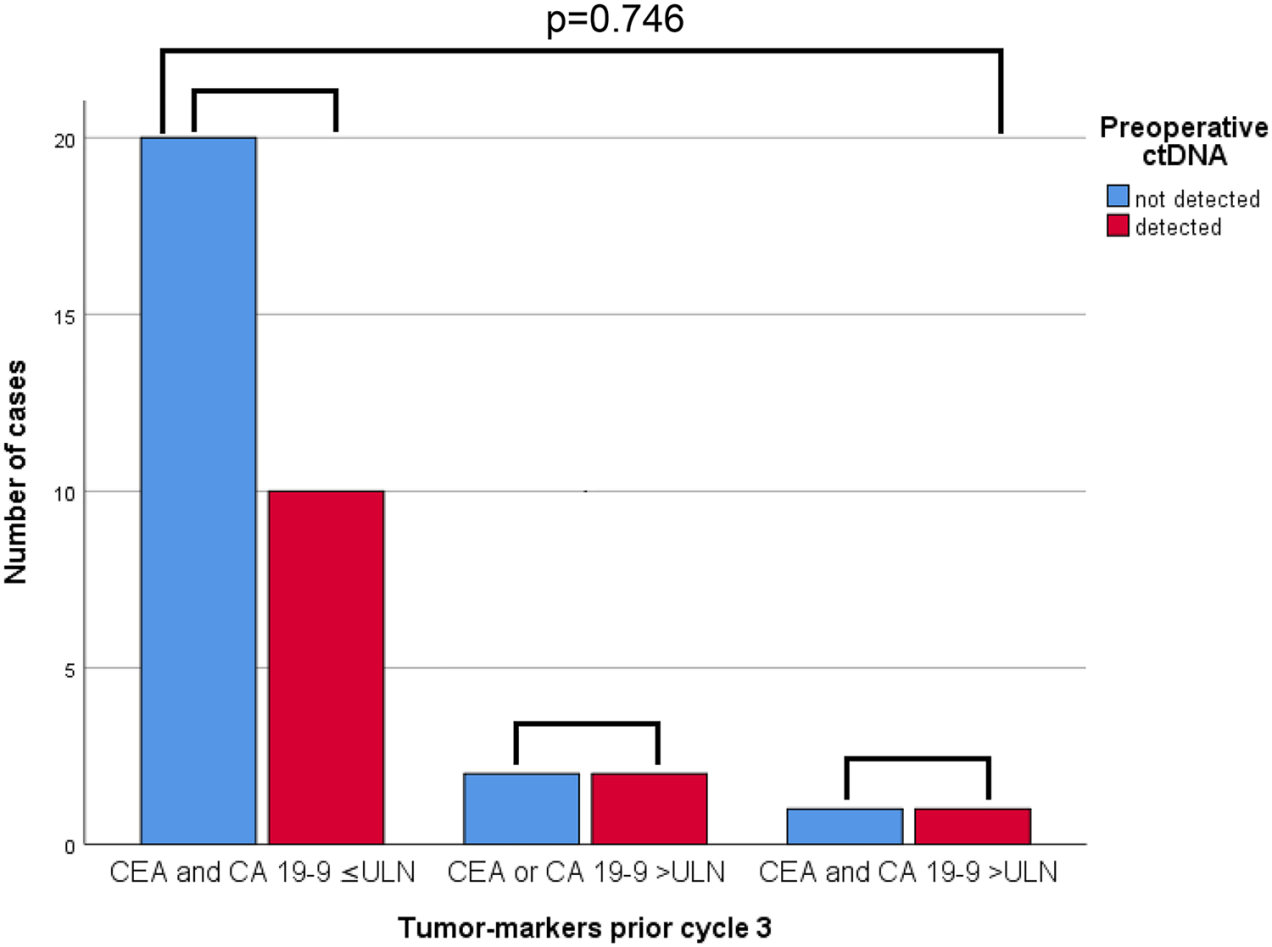
Number of patients with and without detectable ctDNA subdivided by levels of tumor markers (CEA and CA 19-9 within ULN, CEA or CA 19-9 > ULN, or both > ULN)

For 39 out of 50 patients (78%) of whom presence of preoperative ctDNA was determined, information on tumor markers prior to cycle 3 was also available. In this small sample, there was no association between elevated pretreatment tumor markers and presence of ctDNA (details are presented in supplementary Table 4). Although both preoperative presence of ctDNA and tumor markers were prognostic for survival, no association could be confirmed between these two parameters (Fig. 4, supplementary Table 4).

## Discussion

Currently, tumor markers do not play a significant role in treatment decisions for gastric cancer patients without metastatic disease. In this study, we evaluated the prognostic value of blood-derived laboratory parameters including tumor markers, in a European cohort of patients with potentially resectable gastric cancer. We showed that elevated pretreatment levels of CEA and CA 19-9 were associated with worse OS and EFS. In addition, our data suggest that determination of tumor markers at several time-points preoperatively can be helpful in predicting patient outcome. The sample size to evaluate the relationship between tumor markers and ctDNA was limited. No association between those two was detected. These results suggest that future studies to confirm whether both factors independently could be of prognostic value, are needed. In addition, the combination of tumor markers and ctDNA could potentially be valuable in predicting OS and help tailor treatment.

We confirmed CEA and CA 19-9 as prognostic factors for survival in a European cohort of patients with potentially curable gastric cancer. The outcomes of the current study are in line with systematic reviews on the prognostic value of CEA and CA 19-9. A meta-analysis evaluating the prognostic value of CEA included 14,651 mainly Asian patients with gastric cancer treated with either curative or palliative intent and reported shorter disease-free survival (HR 2.28, 95% CI 1.84–2.82) and worse OS (HR 1.63, 95% CI 1.46–1.82) in patients with elevated pretreatment CEA [8]. Although most studies used a cutoff value of 5 µg/L for CEA, in this study we used a cutoff value of 6 µg/L because this cutoff is maintained in Dutch clinical practice and the majority of patients included in the CRITICS trial were Dutch. Another meta-analysis included 11,408 patients with gastric cancer, mainly from Asia, of whom the majority was treated with curative intent and evaluated the prognostic value of CA 19-9 [7]. Elevated pretreatment level of CA 19-9 was associated with a worse OS (HR 1.83, 95% CI 1.56–2.15). Although different cutoffs for CA 19-9 are in use, most studies used—similar to our study—a cutoff value of 37 kU/L. In our study, we reported higher prognostic value of the combination CEA and CA 19-9 as compared to these factors separately, in line with a previous report on esophageal cancer patients [22]. The current study showed that patients in whom both pretreatment tumor markers were elevated had a poor prognosis. Notwithstanding, there is still a proportion of patients in whom long-term survival could be achieved. This indicates that for a subset of gastric cancer patients there is room for treatment intensification.

In clinical practice, tumor markers are often used as surrogate to monitor ‘response’, although this has never been established in patients with resectable gastric cancer. In this study, prognostic value of the change of tumor markers during preoperative therapy has been investigated. As expected, patients who did not have elevated tumor markers at both time-points had the most favorable survival, while patients who had elevated tumor markers at both time-points had the poorest outcome. These results indicate that patients who develop elevated tumor markers during treatment have an unfavorable prognosis, and that patients who started with elevated tumor markers but ‘responded’ on treatment had better survival compared to ‘non-responders’. In addition, we investigated the association between tumor markers and the likelihood to undergo surgery with curative intent. Patients with elevated pretreatment tumor markers were less likely to undergo surgery with curative intent. Tumor marker dynamics during preoperative therapy might help in selecting gastric cancer patients for surgery.

Whereas tumor markers are commonly used in clinical practice, ctDNA is a relatively new research field in patients with cancer. In upper-gastrointestinal cancer, only few small studies have been reported. One study was performed in 45 patients who underwent chemoradiotherapy for localized esophageal cancer [23]. Two other studies have been performed in patients with resectable gastric cancer [19, 24]. The study of Leal et al., based on a subgroup analysis of the CRITICS trial, evaluated the prognostic significance of ctDNA among 50 patients. Baseline detection of ctDNA alone was not prognostic, but when combined with preoperative presence of ctDNA, molecular ‘responders’ and ‘non-responders’ could be identified. Whether or not a patient responded was significantly associated with histopathological response. Postoperative detection of ctDNA was highly associated with recurrence and OS. The same conclusion was made by Yang et al. among 38 patients with resectable gastric cancer. Based on these studies in patients with upper-gastrointestinal cancer, ctDNA is regarded a promising new tool for individualization of treatment in the future.

To further explore the role of ctDNA in gastric cancer, we here compared detection of ctDNA with the presence of elevated tumor markers. No association between ctDNA and tumor markers has been found. To interpret these results, it is important to take the half-life of tumor markers and ctDNA into account. Whereas the half-life of ctDNA is very short—up to several hours—the half-life of tumor markers is much longer, up to several days [25, 26]. There are two important limiting factors of this post-hoc analysis of the CRITICS trial. The timing of determination of tumor markers and ctDNA as per protocol was not defined in advance. Hence, determination of pretreatment tumor markers and ctDNA was not necessarily performed at the same time-points. In addition, preoperative tumor markers were not recorded consistently. Consequently, we chose to compare tumor markers determined prior to cycle 3 of preoperative chemotherapy with ctDNA after cycle 3 of chemotherapy. Another limitation is the small sample size, which is likely to reduce the chance of detecting a true association between tumor markers and ctDNA. However, in gastric cancer, the association between ctDNA and tumor markers has not been explored before and the current findings advocate for future trials to explore this relationship. Thus, we anticipate that the combination of tumor markers and ctDNA could serve as a strong combination of prognostic factors for recurrence and survival, especially preoperatively.

The present study has other limitations, such as the fact that this was a post hoc analysis of the CRITICS trial, and the original statistical plan was not designed and powered for the current analysis. Second, since this was a retrospective analysis determination and collection of blood-derived laboratory parameters including parameters were not obligatory in this study, resulting in an incomplete dataset. In addition, higher levels of CEA and CA 19-9 have been reported in patients with a higher tumor stage [8, 27]. In the current study, baseline stage was not available. However, in models correcting for tumor volume, tumor markers remained prognostic for survival [7, 8].

At this moment, patients with gastric cancer are treated according to a one-size-fits-all principle. The combination of tumor markers and ctDNA could help tailor treatment in the future. There are many other baseline factors and other prognostic factors which could be targets for individualization of therapy in the future. Examples of other factors known at baseline include age, gender, and tumor type based on Lauren classification, human epidermal growth factor receptor-2 (HER-2) status, Epstein–Barr Virus (EBV) status and microsatellite status [28]. Examples of factors known after surgery include tumor regression, lymph node positive disease and resection margin [28, 29]. Prognostic factors are very important to enable individualization of therapy.

In conclusion, CEA, CA 19-9 and ctDNA are valuable prognostic factors. The results described in this study help gain insight in disease patterns of patients with gastric cancer. Our data suggest that future studies should include tumor markers and preferably also ctDNA, for example as stratification factor and as target for clinical decision making.

## Supplementary Information

Below is the link to the electronic supplementary material.

Supplementary file1 (DOCX 15 KB)

Supplementary file2 (DOCX 15 KB)

Supplementary file3 (DOCX 14 KB)

Supplementary file4 (DOCX 13 KB)

Supplementary file5 (DOCX 118 KB)

Supplementary file6 (DOCX 115 KB)

Supplementary file7 (DOCX 112 KB)

Supplementary file8 (DOCX 60 KB)
