# Supplementary material for: Prognostic value of tumor markers and ctDNA in patients with resectable gastric cancer receiving perioperative treatment: results from the CRITICS trial
Source: Gastric Cancer. 2021 Oct 29;25(2):401–10. doi: 10.1007/s10120-021-01258-6 (PMC8882113; doi:10.1007/s10120-021-01258-6)
Supplement: Supplementary file 2 — Supplementary file2 (DOCX 15 KB) [file 10120_2021_1258_MOESM2_ESM.docx]

| **Variable** | **n** | **HR** | **95% CI** | **p value** |
| --- | --- | --- | --- | --- |
| Age in years  <60  60-69  ≥70 | 319 (41%)  297 (38%)  172 (22%) | *  0.935  1.01 | 0.78-1.16  0.81-1.27 | 0.622  0.910 |
| Sex  Male  Female | 529 (67%)  259 (33%) | *  1.06 | 0.88-1.27 | 0.572 |
| WHO PS  0  1 | Missing n=45  534 (72%)  209 (28%) | *  1.54 | 1.27-1.86 | <0.001 |
| Lauren classification (biopsy)  Intestinal Diffuse  Mixed  Unknown | 255 (32%)  235 (30%)  45 (6%)  253 (32%) | *  1.54  0.991  1.18 | 1.24-1.93  0.59-1.39  0.94-1.47 | <0.001  0.655  0.148 |
| Tumour localisation  GOJ  Proximal  Middle  Distal | 142 (18%)  157 (20%)  231 (29%)  258 (33%) | *  1.00  1.04  0.84 | 0.76-1.33  0.80-1.35  0.65-1.08 | 0.985  0.761  0.173 |
| BMI  ≥30  25-30  18.5-25  ≤18.5 | 105 (13%)  276 (35%)  385 (49%)  21 (3%) | *  0.99  1.36  1.28 | 0.74-1.33  1.03-1.79  0.72-2.28 | 0.953  0.030  0.410 |
| Allocated treatment  Postop CT  Postop CRT | 395 (50%)  393 (50%) | *  1.04 | 0.88-1.24 | 0.650 |
| CEA  ≤6 µg/L  >6 µg/L | Missing n=63  604 (83%)  121 (17%) | *  1.63 | 1.30-2.05 | <0.001 |
| CA 19-9  ≤37kU/L  >37kU/L | Missing n=89  531 (76%)  168 (24%) | *  1.76 | 1.43-2.16 | <0.001 |
| CEA + CA 19-9  Both ≤ULN  CEA or CA 19-9 >ULN  Both >ULN | Missing n=98  461 (67%)  177 (26%)  52 (8%) | *  1.47  2.82 | 1.19-1.82  2.06-3.87 | <0.001  <0.001 |
| Alkaline phosphatase  ≤115 U/L  >115 U/L | Missing n=10  734 (94%)  44 (6%) | *  1.11 | 0.76-1.60 | 0.596 |
| Neutrophils  ≤7.5 10^9^/L  >7.5 10^9^/L | Missing n=66  85 (91%)  64 (9%) | *  1.05 | 0.77-1.44 | 0.761 |
| Hemoglobin  ≤10 or 11 mmol/L *  >10 or 11 mmol/L | Missing n=1  367 (47%)  420 (53%) | *  1.07 | 0.90-1.28 | 0.436 |
| LDH  ≤248 U/L  >248 U/L | Missing n=46  659 (89%)  83 (11%) | *  1.18 | 0.90-1.55 | 0.227 |

**Supplementary Table 2:** Univariable analysis of baseline clinical and blood derived laboratory parameters on event-free survival, including tumor markers. *Hemoglobin ≤10 mmol/L for females and ≤11 mmol/L for males.
