# Supplementary material for: Prognostic value of tumor markers and ctDNA in patients with resectable gastric cancer receiving perioperative treatment: results from the CRITICS trial
Source: Gastric Cancer. 2021 Oct 29;25(2):401–10. doi: 10.1007/s10120-021-01258-6 (PMC8882113; doi:10.1007/s10120-021-01258-6)
Supplement: Supplementary file 3 — Supplementary file3 (DOCX 14 KB) [file 10120_2021_1258_MOESM3_ESM.docx]

| **Variable** | **n=650** | **HR** | **95% CI** | **p value** |
| --- | --- | --- | --- | --- |
| Lauren classification  Intestinal  Diffuse  Mixed  Unknown | 200 (31%)  194 (30%)  37 (6%)  219 (34%) | *  1.79  0.86  1.35 | 1.38-2.31  0.52-1.42  1.05-1.74 | <0.001  0.559  0.022 |
| WHO PS  0  1 | 466 (72%)  184 (28%) | *  1.35 | 1.09-1.67 | 0.005 |
| BMI  ≥30  25-30  18.5-25  ≤18.5 | 87 (13%)  235 (36%)  312 (48%)  16 (2%) | *  1.02  1.29  1.02 | 0.73-1.43  0.94-1.77  0.53-1.99 | 0.895  0.112  0.948 |
| Allocated treatment  Postop CT  Postop CRT | 395 (50%)  393 (50%) | *  1.02 | 0.84-1.25 | 0.824 |
| CEA  ≤6 µg/L  >6 µg/L | Missing n=138  543 (84%)  107 (16%) | *  1.58 | 1.23-2.03 | <0.001 |
| CA 19-9  ≤37kU/L  >37kU/L | Missing n=138  499 (77%)  151 (23%) | *  1.79 | 1.43-2.24 | <0.001 |

**Supplementary Table 3:** Multivariable analysis for prognostic factors on event-free survival EFS including tumor markers.
