# Supplementary material for: Prognostic value of tumor markers and ctDNA in patients with resectable gastric cancer receiving perioperative treatment: results from the CRITICS trial
Source: Gastric Cancer. 2021 Oct 29;25(2):401–10. doi: 10.1007/s10120-021-01258-6 (PMC8882113; doi:10.1007/s10120-021-01258-6)
Supplement: Supplementary file 4 — Supplementary file4 (DOCX 13 KB) [file 10120_2021_1258_MOESM4_ESM.docx]

|  | **Variable** | **ctDNA detected** | **ctDNA not detected** | **p value** |
| --- | --- | --- | --- | --- |
| Pretreatment | CEA  ≤6 µg/L  >6 µg/L | 20 (87%)  3 (13%) | 19 (76%)  6 (24%) | 0.466 |
|  | CEA level in µg/L  Median (IQR) | 2.1 (1.2-5.0) | 3.0 (1.3-6.5) | 0.475 |
|  | CA 19-9  ≤37kU/L  >37kU/L | 17 (74%)  6 (26%) | 20 (83%)  4 (17%) | 0.494 |
|  | CA19-9 level in kU/L  Median (IQR) | 9 (6-41) | 14 (2-25) | 0.831 |
| Preoperative | CEA  ≤6 µg/L  >6 µg/L | 11 (73%)  4 (17%) | 22 (92%)  2 (8%) | 0.180 |
|  | CEA level in µg/L  Median (IQR) | 3.3 (1.0-7.0) | 3.0 (2.1-5.0) | 0.898 |
|  | CA 19-9  ≤37kU/L  >37kU/L | 11 (85%)  2 (15%) | 20 (87%)  3 (13%) | 0.999 |
|  | CA19-9 level in kU/L  Median (IQR) | 7 (2-35) | 14 (6-23) | 0.871 |

**Supplementary Table 4:** Association pretreatment tumor markers and ctDNA.
