# Supplementary material for: Prognostic value of tumor markers and ctDNA in patients with resectable gastric cancer receiving perioperative treatment: results from the CRITICS trial
Source: Gastric Cancer. 2021 Oct 29;25(2):401–10. doi: 10.1007/s10120-021-01258-6 (PMC8882113; doi:10.1007/s10120-021-01258-6)
Supplement: Supplementary file 5 — Supplementary file5 (DOCX 118 KB) [file 10120_2021_1258_MOESM5_ESM.docx]

a)


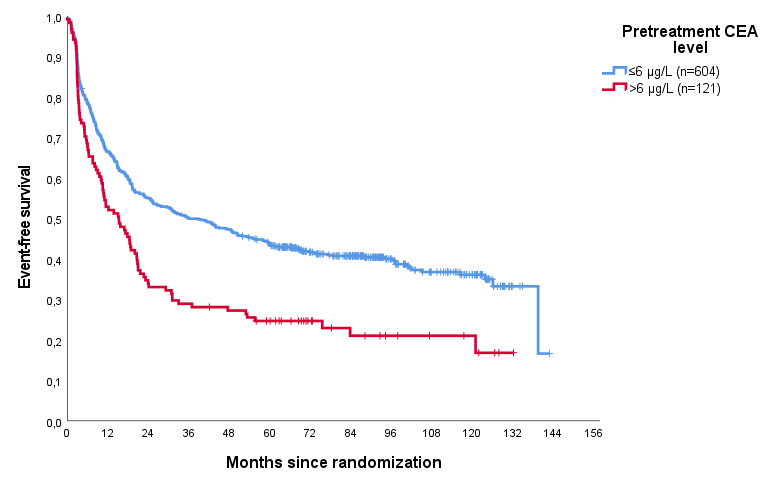


b)


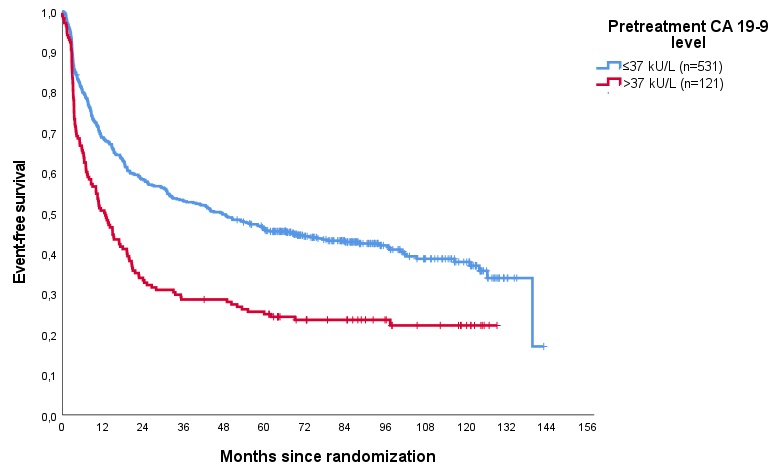


c)
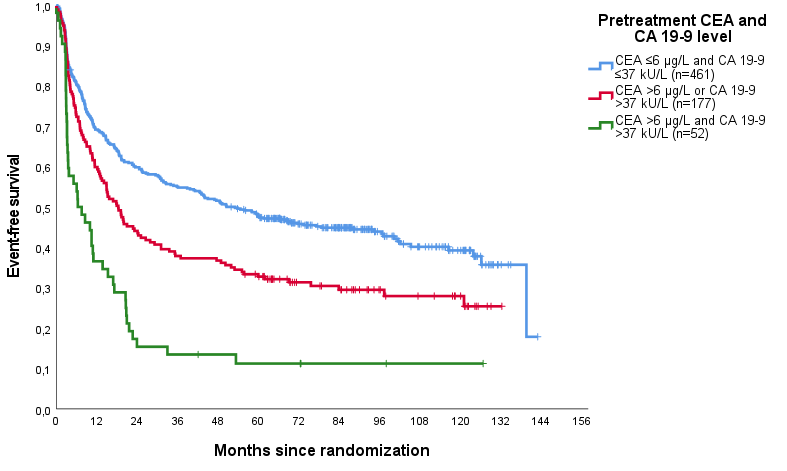


**Supplementary Figure 1:** Event-free survival curves for patients subdivided by pretreatment tumor markers. Figure 1a) CEA (p value <0.001); Figure 1b) CA 19-9 (p value <0.001); Figure 1c) combination CEA and CA 19-9 (p value <0.001)
