# Supplementary material for: Prognostic value of tumor markers and ctDNA in patients with resectable gastric cancer receiving perioperative treatment: results from the CRITICS trial
Source: Gastric Cancer. 2021 Oct 29;25(2):401–10. doi: 10.1007/s10120-021-01258-6 (PMC8882113; doi:10.1007/s10120-021-01258-6)
Supplement: Supplementary file 6 — Supplementary file6 (DOCX 115 KB) [file 10120_2021_1258_MOESM6_ESM.docx]

a)
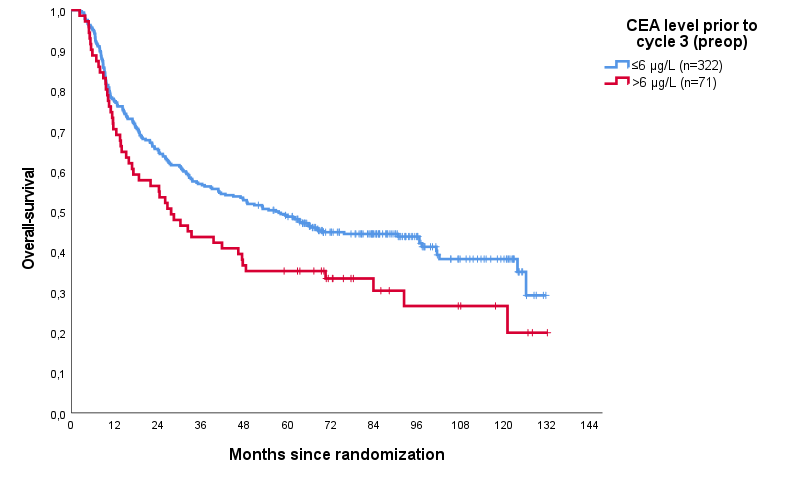


b)


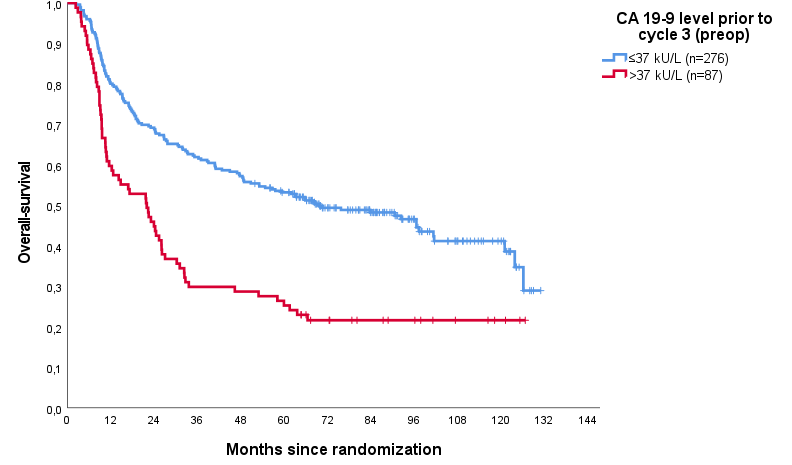


c)
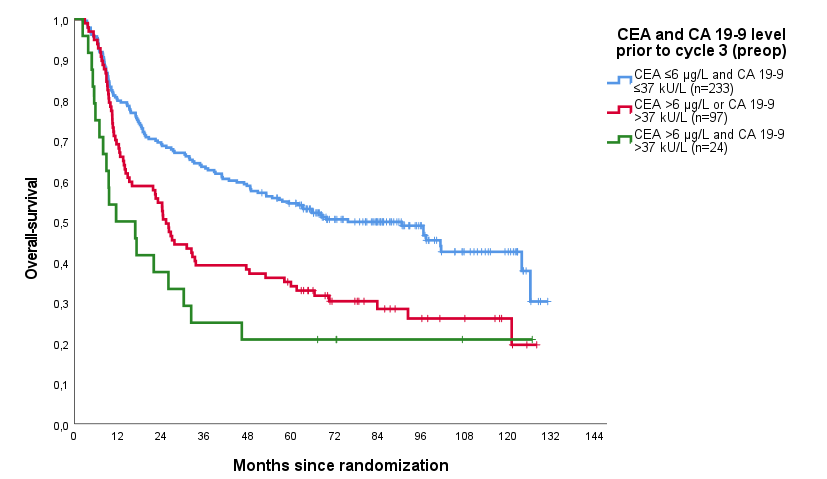


**Supplementary Figure 2:** Overall-survival curves for patients subdivided by tumor markers prior to cycle 3. Figure 2A) CEA (p value=0.030), Figure 2B) CA 19-9 (p value <0.001), Figure 2C) combination of CEA and CA 19-9 (p value <0.001)
