# Supplementary material for: Prognostic value of tumor markers and ctDNA in patients with resectable gastric cancer receiving perioperative treatment: results from the CRITICS trial
Source: Gastric Cancer. 2021 Oct 29;25(2):401–10. doi: 10.1007/s10120-021-01258-6 (PMC8882113; doi:10.1007/s10120-021-01258-6)
Supplement: Supplementary file 8 — Supplementary file8 (DOCX 60 KB) [file 10120_2021_1258_MOESM8_ESM.docx]

**
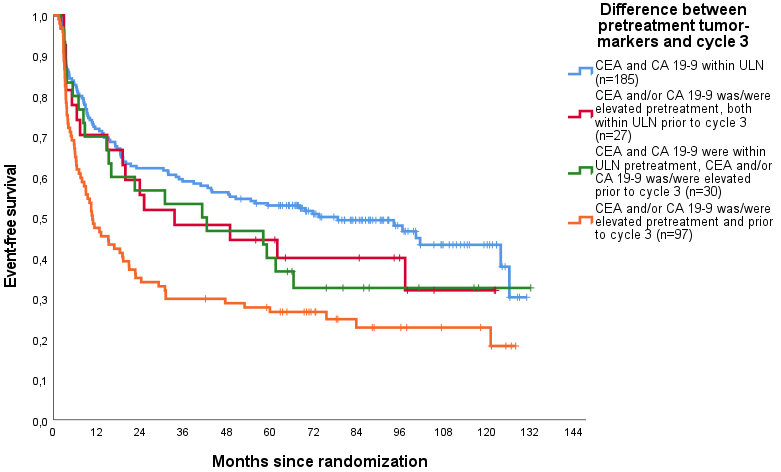
**

**Supplementary Figure 4:** Event-free survival curves for patients subdivided by change between pretreatment tumor markers and tumor markers prior to cycle 3 (p value <0.001)
